# Ethics of HIV cure research: an unfinished agenda

**DOI:** 10.1186/s12910-021-00651-1

**Published:** 2021-06-30

**Authors:** Karine Dubé, John Kanazawa, Jeff Taylor, Lynda Dee, Nora Jones, Christopher Roebuck, Laurie Sylla, Michael Louella, Jan Kosmyna, David Kelly, Orbit Clanton, David Palm, Danielle M. Campbell, Morénike Giwa Onaiwu, Hursch Patel, Samuel Ndukwe, Laney Henley, Mallory O. Johnson, Parya Saberi, Brandon Brown, John A. Sauceda, Jeremy Sugarman

**Affiliations:** 1 University of North Carolina at Chapel Hill, Gillings School of Global Public Health, 4108 McGavran-Greenberg Hall, Chapel Hill, NC 27599-7469 USA; 2 HIV + Aging Research Project – Palm Springs (HARP–PS), Palm Springs, CA USA; 3 AntiViral Research Center (AVRC) Community Advisory Board (CAB), San Diego, CA USA; 4 Collaboratory of AIDS Researchers for Eradication (CARE) CAB, Chapel Hill, NC USA; 5 AIDS Action Baltimore, Baltimore, MD USA; 6 Delaney AIDS Research Enterprise (DARE) Community Advisory Board (CAB), San Francisco, CA USA; 7 BEAT-HIV Collaboratory CAB, Philadelphia, PA USA; 8 defeatHIV CAB, Seattle, WA USA; 9 AIDS Clinical Trials Group (ACTG) Community Scientific Subcommittee (CSS) Ethics Working Group, Nationwide, USA; 10 AIDS Clinical Trials Group Global CAB, Washington, D.C. USA; 11 Institute of Global Health and Infectious Diseases HIV Treatment and Prevention CAB, University of North Carolina at Chapel Hill, Chapel Hill, NC USA; 12 Charles R. Drew College of Medicine and Science, Los Angeles, CA USA; 13 Center for the Study of Women, Gender, and Sexuality (School of Humanities), Rice University, Houston, TX USA; 14 Center for AIDS Prevention Studies (CAPS), Division of Prevention Sciences, UCSF, San Francisco, CA USA; 15 Department of Social Medicine, Population and Public Health, Center for Healthy Communities, University of California, Riverside, Riverside, CA USA; 16 Johns Hopkins Berman Institute for Bioethics, Baltimore, MD USA

**Keywords:** HIV cure research, Research ethics, Experimental medicine, People living with HIV

## Abstract

**Background:**

The pursuit of a cure for HIV is a high priority for researchers, funding agencies, governments and people living with HIV (PLWH). To date, over 250 biomedical studies worldwide are or have been related to discovering a safe, effective, and scalable HIV cure, most of which are early translational research and experimental medicine. As HIV cure research increases, it is critical to identify and address the ethical challenges posed by this research.

**Methods:**

We conducted a scoping review of the growing HIV cure research ethics literature, focusing on articles published in English peer-reviewed journals from 2013 to 2021. We extracted and summarized key developments in the ethics of HIV cure research. Twelve community advocates actively engaged in HIV cure research provided input on this summary and suggested areas warranting further ethical inquiry and foresight via email exchange and video conferencing.

**Discussion:**

Despite substantial scholarship related to the ethics of HIV cure research, additional attention should focus on emerging issues in six categories of ethical issues: (1) social value (ongoing and emerging biomedical research and scalability considerations); (2) scientific validity (study design issues, such as the use of analytical treatment interruptions and placebos); (3) fair selection of participants (equity and justice considerations); (4) favorable benefit/risk balance (early phase research, benefit-risk balance, risk perception, psychological risks, and pediatric research); (5) informed consent (attention to language, decision-making, informed consent processes and scientific uncertainty); and (6) respect for enrolled participants and community (perspectives of people living with HIV and affected communities and representation).

**Conclusion:**

HIV cure research ethics has an unfinished agenda. Scientific research and bioethics should work in tandem to advance ethical HIV cure research. Because the science of HIV cure research will continue to rapidly advance, ethical considerations of the major themes we identified will need to be revisited and refined over time.

## Background

There have been remarkable scientific advancements in the prevention and treatment of HIV infection [1]. Nevertheless, the pursuit of a cure for HIV is now a high priority for researchers, funding agencies, governments and people living with HIV (PLWH) for a variety of reasons: the need for lifelong treatment of HIV infection, cumulative toxicities of antiretroviral therapy (ART), adherence challenges, the costs of ART, barriers to accessing ART, and HIV-related social stigma and discrimination [2–4]. By HIV “cure,” we mean a regimen or intervention capable of either completely eliminating HIV from the body or inducing a state of durable, ART-free virologic suppression in which small quantities of HIV remain but do not actively increase or cause immunological damage. To date, over 250 biomedical studies worldwide are or have been related to discovering a safe, effective, and scalable HIV cure [5].

While promising, such research is associated with a range of ethical considerations [6, 7]. Akin to other early phase research, there have generally been asymmetric benefit/risk profiles in early-phase HIV cure research, with individual research participants bearing the clinical and psychosocial risks while science and society almost solely reaping the benefits [7, 8]. This is particularly the case for research involving risky study procedures, such as analytical treatment interruptions (ATIs) where ART is paused during the research. ATIs are currently necessary to determine whether investigational interventions achieved their intended effects of durable virologic suppression in the absence of ART [9]. In addition to the potential clinical risks to participants, studies employing ATIs may also increase risks to sexual partners of research participants [10–15].

Emanuel and colleagues identified seven requirements for ethical clinical research: (1) *social value*, (2) *scientific validity*, (3) *fair participant selection*, (4) *favorable benefit/risk ratio*, (5) *independent review*, (6) *informed consent*, and (7) *respect for enrolled participants* [9]. Lo and Grady subsequently specified these ethical requirements for HIV cure research [6], while also articulating the need for *collaborative partnerships* with, and having *respect for affected and vulnerable communities*. Sugarman later expanded these ethical considerations to capture the importance of protecting confidentiality, conflict of interest management (both financial and non-financial), and responsible communication of scientific advancements [16].

Substantial empirical and conceptual bioethics scholarship has followed. As described below, most empirical inquiry in HIV cure research ethics has centered around decision-making and informed consent among potential participants [17, 18], as well as the assessment of acceptable risks and benefits of this research [8, 19–23]. Other aspects that have been examined include scientific uncertainty [24], the role of inclusion benefits [25, 26], incentives [27], social value [3, 28, 29], the need to leverage developments in HIV prevention, treatment, and cure research [30, 31], and the meaningful engagement and involvement of PLWH [32–35].

In this paper, we review recent findings and developments in HIV cure research ethics. Based on a scoping review of the literature augmented by input from community advocates involved in HIV cure research, we highlight areas that warrant further ethical inquiry or guidance.

## Methods

We conducted a scoping review of the HIV cure research ethics literature from July to October 2020 and May 2021. The purpose of this review was to obtain a broad perspective on this specific topic, focused on pivotal literature in the field, and was not intended to provide a systematic synthesis. This type of review is ideal when there is a need to organize emerging information about a topic by mapping the literature to inform potential future research [36, 37].

**Fig. 1:**
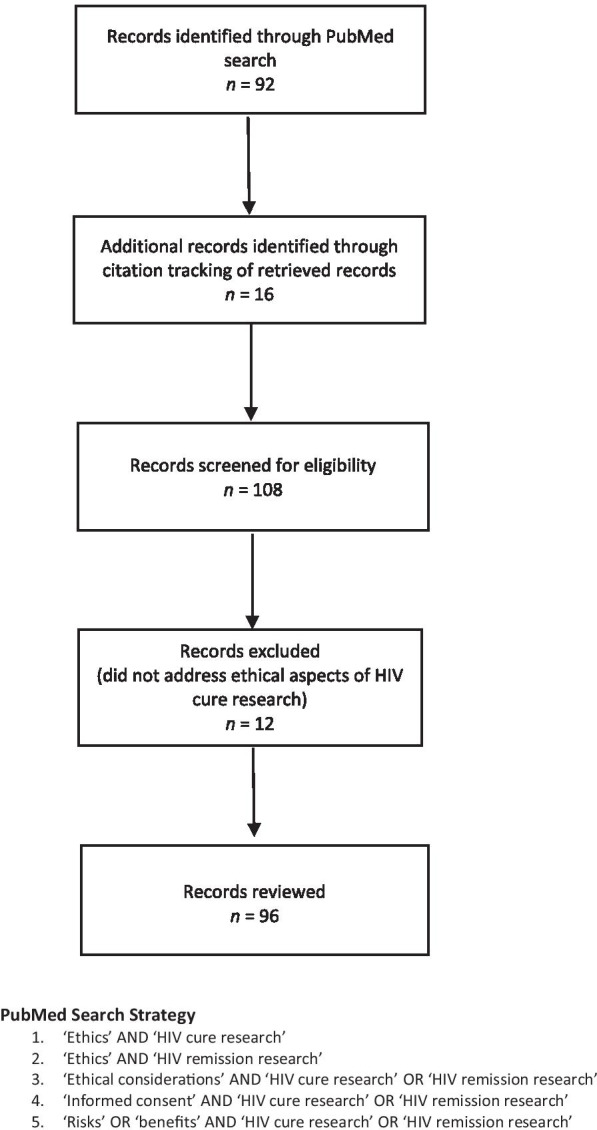
PRISMA-ScR flow diagram: ethics of HIV cure-related research (2013–2021)

Our work generally followed the PRISMA extension for Scoping Reviews (PRISMA-ScR) framework [36]. We concentrated our review on peer-reviewed articles published in the English literature from 2013 to 2021. The year 2013 was selected as a baseline since it corresponds to the publication of the Lo and Grady manuscript on ethical points to consider in HIV cure research [6], which is when other literature related to the ethics of HIV cure research began to emerge. We used PubMed to initially identify articles on the ethical aspects of HIV cure research. The initial search was conducted in July 2020 using the following specific search terms: ‘ethics’ AND ‘HIV cure research’; ‘ethics’ AND ‘HIV remission research’; ‘ethical considerations’ AND ‘HIV cure research’ OR ‘HIV remission research’; ‘informed consent’ AND ‘HIV cure research’ OR ‘HIV remission research’; and ‘risks’ OR ‘benefits’ AND ‘HIV cure research’ OR ‘HIV remission research’. This resulted in 92 articles. We subsequently used citation tracking, pursuing promising references from the articles identified in the initial PubMed searches, adding 16 articles for further consideration. Consistent with the scoping review methodology [36], we did not employ strict criteria for adjudicating the literature. Articles were selected because they specifically addressed ethical aspects of HIV cure research. After removing duplicates and excluding articles that did not address ethical aspects of HIV cure research, we screened and reviewed 96 articles. Figure 1 provides a flow diagram summarizing the process.

In reviewing articles, we extracted and organized ethics findings via manual charting. We used the 2013 Lo and Grady framework [6] to organize the results. Most of the literature we identified fell under six of the eight ethical points to consider in that framework: (1) *social value*; (2) *scientific validity*; (3) *fair selection of participants*; (4) *favorable benefit/risk balance*; (5) *informed consent*; and (6) *respect for enrolled participants and communities*. We did not identify much relevant literature directly related to the other two points of the framework, *collaborative partnership* and *independent review*. The initial scoping review was prepared by a team of core reviewers (K.D., J.K., M.O.J, P.S., B.B., J.A.S).

We circulated the draft scoping review to twelve community advocates (J.T., L.D., N.J., C.R., L.S., M.L., J.K., D.K., O.C., D.P., D.M.C., M.G.O.) for discussion and comment. Community advocates were selected on the basis of being actively engaged in HIV cure research and providing input on clinical trial protocols as part of the Martin Delaney Collaboratory and AIDS Clinical Trial Groups (ACTG) community advisory boards. Community advocates were asked to indicate areas that they considered important as well as suggest areas for which they would like additional guidance on. Community advocates were also consulted via virtual video conferencing in November–December 2020, without the use of a standard guide. Community comments and the resulting manuscript were circulated and discussed via email until all community advocates agreed with the points discussed and the areas that needed further ethical insight.

## Discussion

We identified relevant literature under the following themes: (1) social value (ongoing and emerging biomedical research and scalability considerations); (2) scientific validity (study design issues, such as the use of analytical treatment interruptions and placebos); (3) fair selection of participants (equity and justice considerations); (4) favorable benefit/risk balance (early phase research, benefit-risk balance, risk perception, psychological risks, and pediatric research); (5) informed consent (attention to language, decision-making, informed consent processes and scientific uncertainty); and (6) respect for enrolled participants and community (perspectives of people living with HIV and affected communities and representation).

Table 1 includes outstanding research ethics questions related to these themes.

**Table 1:** Open Research Ethics Questions for HIV Cure Research

**Social Value** *Ongoing and Emerging Biomedical Research* • What are ethical considerations for specific HIV cure research approaches (e.g., cell and gene therapy, immune-based approaches, latency-reversing agents, stem cell transplants, etc.)? • What are ethical considerations for combining HIV cure research regimens? • What are the ethical considerations of conducting HIV cure research with PLWH at the end-of-life—including interventions? • How do we adapt ethical guidelines for such rapidly evolving scientific field? *Scalability Considerations* • What additional ethical considerations are relevant when implementing HIV cure trials in resource-limited settings?	
**Scientific Validity** *Study Design Considerations: Analytical Treatment Interruptions* • When are extended ATIs that may trigger extended periods of viremia ethically appropriate? • What are robust yet practical ethical considerations for mitigating risks to sexual partners of ATI trial participants in diverse circumstances (e.g., anonymous partners, domestic violence potential, access to PrEP/condoms, local criminalization laws)? *Use of Placebos* • When are placebo arms ethically acceptable in HIV cure research?	
**Fair Selection of Participants** *Equity and Justice Considerations* • How can HIV cure research be implemented utilizing ethical principles of equity, social justice, and solidarity?	
**Favorable Benefit/Risk Balance** *Early-Phase Research Considerations* • What are the criteria for judging the ethical permissibility of early-phase HIV cure trials? • How can we ensure trial participants are protected from excessive risks? • How should we navigate the issue of therapeutic ambiguity as HIV cure trials start showing signals of potential efficacy? *Additional Benefit/Risk Considerations* Acceptable Benefit-Risk Balance • What are practical and innovative methods to evaluate acceptability of risk thresholds for emerging HIV cure interventions? • How do we continue to engage relevant stakeholders around the notion of unacceptable risks? • What is the best way to establish additional safeguards for emergent situations that may alter the risk balance (e.g., SARS-CoV-2 pandemic)? Risk Perceptions • What risk thresholds are ethically permissible in different populations of PLWH? • What are ethical considerations and acceptable risks related to extended ATIs requiring PLWH forego ART for extended periods of time? Psychosocial Benefits and Harms • What are the psychosocial benefits of HIV cure research participation, and how should these shape benefit/risk evaluations? • What are some of the non-clinical harms (e.g., psychological, social, legal, and financial) of HIV cure research participation, and how should these shape benefit/risk evaluations? Pediatric HIV Cure Research • How should we evaluate interventional pediatric HIV cure research to ensure acceptable benefit-risk?	
**Informed Consent** *Language Considerations* • What are innovative ways to engage participants, especially diverse communities, around the ethical use of language to describe HIV cure research? *Decision-Making* • What would be the best ways to facilitate participant decision-making in early-phase HIV cure trials? • How should research teams measure therapeutic (or curative) misconception and misestimation in HIV cure trials? *Informed Consent Processes* • What would be the best ways to enhance the quality of informed consent in early-phase HIV cure trials? • Should a separate category of scientific uncertainty disclosure be mandated as part of regulations (i.e., Common Rule)? *Scientific Uncertainty* • What are ethical considerations for communicating scientific uncertainty?	
**Respect for Enrolled Participants and Communities** *Perspectives of PLWH and Affected Communities* • How can the perspectives of PLWH and HIV care providers help prioritize interventions under development to augment their acceptability? *Community Representation* • What are ethical considerations necessary for meaningfully engaging all stakeholders, including patients, providers, government, and the overall community in early-phase HIV cure research?	

### *Social value*: ongoing and emerging biomedical research and scalability considerations

*Ongoing and Emerging Biomedical Research*: HIV cure research involves a variety of biomedical approaches, including cell and gene therapies [38], stem cell transplants [39], immune-based strategies [40, 41], early and intensified HIV therapy [42], and latency-reversing agents [43, 44] among others. These strategies can also be used in combination. Each HIV cure research approach has its own unique ethical considerations and these need to be taken into account to assess their ethical acceptability [45]. For instance, Sugarman outlined considerations for when ATIs following stem cell transplantation should be ethically permissible [46]. As HIV cure clinical trials are implemented, Institutional Review Boards (IRBs), and their functional equivalents, need to be prepared to evaluate HIV cure research protocols to ensure they are ethically acceptable [47]. More deliberation may be needed to create guidelines to inform the ethical evaluation of distinct and emerging HIV cure research approaches.

Moreover, an effective HIV cure may require a combination of approaches which has the potential to substantially increase clinical risks compared to standard ART [48, 49]. For example, some biomedical HIV cure research is investigating whether two or more interventions with multimodal activity may help deplete persistent HIV reservoirs and strengthen immunity [50]. Given the complex nature of latent, integrated HIV proviruses [43], single interventions may not alone result in effective disruption of latency or durable virologic suppression without ART [2, 50]. Some biomedical scientists are even asking whether single interventional HIV cure trials should be completely abandoned [50]. One proposal is to move directly and efficiently from in vitro studies into combination therapy trials in animals, and then into human testing [50].

Further attention is needed to anticipate the challenges related to the ethical implementation of combinatorial HIV cure strategies. Examples include combination gene-editing approaches [51], latency-reversing agents in synergy with immune-modifying agents [43, 48, 49, 52, 53], or different permutations of broadly-neutralizing antibodies paired with different HIV cure approaches [54, 55]. There are now over 20 active HIV cure clinical studies using combination products with intersecting mechanisms underway in the United States alone [5]. Combination HIV cure regimens will likely involve additive, synergistic, or antagonistic effects; these combinatorial effects will affect how benefit/risk assessments are performed. Their efficiency and feasibility will rest upon the appropriate ethical and regulatory reviews/approval processes. Ensuring that PLWH, providers, and other stakeholders view these complex regimens as acceptable will be critical (discussed further below).

There is no doubt that the field of HIV cure research is rapidly evolving, and that the related ethics considerations must be relevant to these scientific developments. For example, a novel approach has been HIV cure-related research at the end of life. For example, a research cohort has been assembled in the “Last Gift Study” [56] that includes PLWH who are terminally-ill with a co-morbid condition (e.g., cancer, advanced heart disease, neurodegenerative disease) and seeks to characterize HIV reservoirs in the brain and deep tissues [57–59]. Drawing on a similar paradigm in the cancer field [60], a multi-disciplinary group of researchers outlined ethical considerations for conducting HIV cure research with PLWH at the end of life [57], including: (1) protecting autonomy through informed consent; (2) avoiding exploitation by fostering altruism; (3) preserving favorable benefit/risk balance; (4) safeguarding against vulnerability through participant-centeredness; and (5) involving next-of-kin/loved ones and community stakeholders [57, 61–63]. Further, testing HIV cure research interventions in PLWH at the end of life would introduce important ethical complexities since terminally ill PLWH would undergo potentially risky interventions solely to advance science, and not in the hope of alleviating symptoms or prolonging life [57, 64]. More work is needed to guide ethics reviews of such novel protocols.

*Scalability Considerations*: Bioethicists, researchers, and community members have recognized that scalability of interventions is potentially a key factor by which to judge the social value of HIV cure research strategies [3, 7, 28, 29, 45, 65, 66]. This aligns with the rationale for advancing translational research related to an HIV cure [45, 67, 68]. That is, successful HIV cure modalities should ultimately be translatable from “bench to patients” and be applicable to diverse clinical care settings and populations around the world [45]. Consequently, to enhance social value, it is essential to consider the ethical and practical challenges related to translating these interventions into real-world settings [45].

Globally, only an estimated 60% of PLWH are on ART, and the financial sustainability of HIV treatment programs remain uncertain [29]. A modeling study conducted in South Africa estimated that an HIV cure would have the greatest impact on HIV incidence if the HIV epidemic is not mitigated by 2030, and that an HIV cure should be prioritized for those not able to access ART and achieve viral suppression [69]. The Bill and Melinda Gates Foundation is engaging industry, government, academic, and community leaders to define a target product profile for a globally scalable HIV cure [66]. Increased knowledge related to efficacy, toxicity, design, delivery, durability, follow-up, relapse, participant-perceived benefits and risks, and cost-effectiveness will increase the likelihood that an HIV cure research strategy can become a viable option on a global scale [3, 29, 66, 70]. It will be desirable to predict and essential to precisely and quickly detect loss of viral suppression and resistance in both resource-rich and resource-limited settings [66]. Challenges related to clinical and laboratory capacity, financing, training, distance to health care providers, and health systems also need to be overcome [45]. Biological factors such as prevalence of co-occurring conditions (such as the presence of other infectious or non-communicable diseases, and poor nutrition) and differing HIV subtypes will also need to be considered [28]. To enhance social value, studies will need to be conducted in communities that would most likely benefit from an HIV cure [29] and successful interventions should become available to the populations that faced the risks of testing the intervention [39, 71, 72]. HIV cure research developments will also need to be synergistically integrated with ongoing HIV prevention and treatment efforts [30, 31, 45, 73].

### *Scientific validity*: study design considerations—ATIs and use of placebos

*Analytical Treatment Interruptions*: ATIs remain one of the most controversial topics in HIV cure research, involving considerations for medical, research, and public health ethics [9, 45, 74–76]. In addition to presenting risks to study participants, such as developing acute retroviral syndrome and/or HIV resistance, ATIs present risks of HIV transmission to the sexual partners of participants [15, 77–79]. Following two cases of HIV transmission in the context of an ATI [14, 15, 78], a risk mitigation plan was developed to ensure ATIs could be conducted more safely and ethically. This plan included an ATI study disclosure checklist separate from the consent form and pre-exposure prophylaxis (PrEP) navigation resources for partners [13]. Given the importance placed by many PLWH on becoming and remaining undetectable for HIV so that they do not transmit HIV to partners [80–82], understanding the ethical challenges related to mitigating risks during ATIs is of paramount importance [83]. Determining appropriate risk mitigation strategies will further require effective stakeholder engagement in local contexts [84–86]. Developing self-administered point of care rapid tests to detect and measure viral rebound with clearly delineated ART restart criteria would also help to mitigate risks to participants and the risk of onward HIV transmission during ATIs.

*Use of Placebos*: As in other research settings, determining the ethical and scientific appropriateness of placebo-controlled trials in HIV cure research can be complex [7, 9]. After extensive deliberation, a multi-disciplinary group of HIV cure experts concluded that placebo arms should be used where necessary and appropriate to confirm scientific validity of trials [9]. Moreover, they contended it would be unethical not to use placebo groups when scientifically necessary [9]. In early exploratory ATI studies, scientists have opted to use historical controls to compare time to HIV rebound [9, 87]. To minimize risks, another option would be to establish a placebo cohort that could serve as control for multiple trials occurring contemporaneously. Of note, PLWH may be reluctant to enroll in placebo-controlled trials and go off ART for extended periods of time while not knowing whether they received an experimental intervention [9, 75, 88].

### *Fair selection of participants*: equity and justice considerations

*Equity and Justice Considerations*: Diversity in clinical trials is a matter of justice [45]. Populations that may benefit the most from an HIV cure are most often unable to participate or are excluded from clinical trials [20, 45]. For HIV cure research, efforts should be made to increase representation of cis- and transgender women, as well as other gender diverse individuals, people of color, people who do not speak English as their primary language, and other under-represented and marginalized groups [89]. For the HIV cure research enterprise to be truly transformative and forward-looking, research teams will need to be committed to social justice, racial and gender equity, and solidarity in research activities. Further, partner risk mitigation strategies during ATIs, such as the use of PrEP, must be acceptable to diverse populations. Such plans must also be appropriate with respect to gender and sex dynamics, accounting for issues related to stigma, the potential need to incorporate trauma-informed approaches [90] and the potential for intimate partner violence [13, 91].

### *Favorable benefit/risk balance*: early-phase research and additional benefit/risk considerations

*Early-Phase Research Considerations*: HIV cure research harbors similar ethical challenges to early-phase trials in other areas of medicine [92]. Yet unlike early-phase cancer trials in which study participants may be terminally ill or otherwise have a poor prognosis, most HIV cure study participants include “otherwise healthy volunteers” who are on highly effective ART [20]. Current early-phase HIV cure trials represent the inverse of the early years of the HIV epidemic when PLWH’s best chance for survival often meant undertaking significant risk through unproven therapies or participation in early phase treatment trials [23]. Against a backdrop of highly effective and well-tolerated therapies, current safety thresholds for moving novel anti-HIV therapies forward have become extremely high [20, 23]. Additionally, many early-phase HIV cure studies are exploratory with little or no likelihood of being curative or providing direct benefit to participants [19]. Moreover, there are currently no established standards for assessing the ethical permissibility of risks in early-phase HIV cure trials, especially when novel strategies rely solely on pre-clinical evidence (e.g., data from prior cell and animal studies) to initiate human testing [93].

The ethical appropriateness of early phase HIV cure research typically relies on the judgments and abilities of researchers, IRBs, and regulatory agencies. In evaluating the ethical permissibility of early-phase HIV cure trials, the risks of interventions and ATIs must be considered along with proposed monitoring procedures and risk mitigation strategies (e.g., when ART resumption is warranted following an ATI).

Another challenge for early-phase HIV cure trials relates to the extent to which participants are motivated by altruism [94]. When participation is motivated by altruism, participants may be willing to accept greater risk, thus lessening concern regarding potential exploitation [57, 95].

Further, uncertainty about whether and when an intervention will have a sufficient intended effect to advance further studies, is an area that needs further attention. Specifically, what are the relevant considerations in determining if it is appropriate to advance particular interventions into larger human trials? Currently, there is no consensus regarding what constitutes sufficient benefits for an intervention to be considered “curative.”

*Additional Benefit/Risk Considerations*: There are a variety of issues related to benefit/risk considerations in the ethics literature on HIV cure research: acceptable benefit/risk balance, risk perceptions, psychosocial benefits and harms, and those particular to pediatric HIV cure research.

*Acceptable Benefit/Risk Balance*: Major research guidelines require that clinical trials maintain an acceptable benefit/risk balance [8, 96, 97], yet limited concrete and practical guidance exists to help ensure this requirement is met [98–100]. Maintaining an acceptable benefit/risk balance is necessary because it not only minimizes harming participants, but also helps protect researchers’ professional integrity and maintain public trust in science [23, 98, 101, 102]. In most early-phase HIV cure research, the ethical analysis involves a *knowledge-risk calculus* [8, 103] that evaluates whether the potential scientific knowledge and additional data justify the risks. HIV cure research walks a fine line between the safety and tolerability of experimental interventions, and the potential efficacy from an intervention [20]. Steel argues that regulatory limits on risks are *prima facie* paternalistic [104]. To the contrary, Różyńska argues that upper risk limits are justified to protect both the research enterprise and participants from unjustified and excessive risks because of inherent power inequities between investigators and participants [102]. Thus, there is a need for further deliberation about the acceptable level of risks in this research.

A substantial amount of scholarship has focused on the benefits and risks of HIV cure research [8, 96, 105–107]. Eyal summarized various candidate solutions to keep high-risk HIV cure studies ethical, such as reducing risks through robust pre-clinical data, dose limits, and patient engagement, enhancing benefits to individual participants by making participation appealing, recognizing psychosocial benefits of participation, and building towards societal benefits [105]. As an example, one research team was able to enhance the benefits of an HIV cure study and augment the clinical care of participants by offering colon cancer screening alongside invasive gut biopsy procedures [108]. Another proposal by Largent [106] suggested offering payments to augment the benefit/risk profile.

In evaluating benefit/risk favorability, one must consider the types of interventions, the anticipated risks, the background standard of care, and the health status of trial participants [22]. Nevertheless, some risks will be difficult, if not impossible, to capture in short-term clinical trials [38]. For example, some trials may carry long-term toxicity risks such as teratogenicity and gene toxicity that could manifest years after a trial has been completed [20, 94]. For most interventions, the United States Food and Drug Administration (FDA) has considered PLWH as “otherwise healthy volunteers” to ascertain acceptable risks, thereby decreasing the threshold of risk that could be tolerated in this population [22, 23]. DiGiusto and colleagues described five additional categories of participants in HIV cure cell and gene therapy research: (1) PLWH with significant ART side effects and “treatment fatigue;” (2) PLWH not virally suppressed and with incomplete immune recovery; (3) ART non-responders; (4) PLWH with cancer; and (5) PLWH requiring salvage therapy or cancer treatment (e.g., transplants) [39]. If participants are not “otherwise healthy,” this may increase the level of risks that could be tolerated in these populations [20]. For example, while a stem cell transplant would be too risky an undertaking for an “otherwise healthy” individual, it may be justifiable as a means of attempting to cure HIV in a person who is already in need of a transplant to treat their cancer. This strategy was used for two people who were cured of HIV: the late Timothy Ray Brown, known as the “Berlin patient”, who died of cancer more than 12 years after his HIV cure; and Adam Castillejo, sometimes referred to as the “London patient” [109, 110].

Increasing attention is being paid to the role of safeguards needed to protect study participants in HIV cure trials to minimize risks [9, 20, 23, 75, 111]. Maximizing safety while minimizing burdens of monitoring visits (e.g., frequent viral load testing during ATIs) is another fundamental tension in clinical trial design [75]. Added protections have included robust deliberations around trial design [9], participant selection and inclusion/exclusion criteria [9, 76], informed consent [9, 17, 76], monitoring and safety rules [9], ART restart criteria in the case of ATIs [9, 76], and involvement of community advisory boards (CABs) [6]. Further, as in most clinical research, emergent situations such as the SARS-CoV-2 pandemic precipitate the need for additional safeguards to protect trial participants from undue harm [112, 113]. Safeguards are also essential in trials requiring ATIs. Two independent systematic reviews [114, 115] showed that brief ATIs (e.g., a few weeks in duration) did not present substantial risk of adverse events (AEs). However, much less is known about the risks involved in extended ATIs (e.g., a few months), involving sustained periods of viremia [9]. To properly evaluate extended ATIs, it is imperative to understand not only their clinical risks, but also their psychological and social risks.

*Psychosocial Benefits and Harms*: HIV cure research has also been associated with psychosocial benefits that have been captured through empirical assessments [25, 107, 116–118] and testimonials [119–121]. These psychosocial benefits include improved sense of purpose, positive outlook, hope, and emotional support [26]. Understanding the altruism among many HIV cure trial participants helps contextualize the impact of trial participation on people’s lives [94, 107]. Providing an activist’s argument that participant values should guide benefit/risk ratio calculations, Evans claims these altruistic benefits should be recognized because they empower PLWH to assume both self-agency and autonomy as well as risks [107]. In turn, Rennie and colleagues argued that these collateral psychosocial benefits align with the principle of beneficence and advocate for more detailed guidance on how to account for these benefits in regulatory reviews, consent documents, and trial communications [25, 26].

Psychological harms include negative mental states and anxiety, such as the fear of developing drug-resistant HIV or passing HIV on to sexual partners as a result of no ART during ATIs [15, 75, 77, 81, 107, 119]. Social harms encompass the risks of disrupting one’s social network and/or increasing stigmatization because of trial participation [100]. Further, ATIs may also cause confusion by contradicting long-standing messages from HIV care teams regarding the need for sustained ART adherence [75, 80, 81]. Due to some existing HIV criminalization statutes, PLWH may also face legal ramifications should they engage in behavior perceived as likely to transmit HIV to another party during an ATI [75]. The potential psychological and mental health dimensions of taking part in intensive HIV cure trials should not be minimized, especially protocol designs requiring participants to be off ART for extended periods of time [70, 112]. Given the enduring social stigmatization of HIV, both conventional clinical as well as psychological risks should be integrated into potential study benefit/risk calculations.

*Risk Perceptions*: A body of empirical research has ascertained stakeholder perceptions of acceptable risk thresholds [19, 20, 88, 122]. This research has revealed disagreements between stakeholder types (e.g., PLWH, regulators, bioethicists, biomedical researchers, and clinicians) regarding what constitutes “too much risk” in HIV cure research [20]. As the science evolves, the standard of acceptability may shift and clinical risks may become better defined [9, 21]. IRBs may also weigh potential benefits and risks differently [20]. Therefore, some IRBs may allow trials with extended ATIs resulting in sustained periods of viremia to move forward, while others may prevent these trials from proceeding.

*Pediatric HIV Cure Research*: Pediatric HIV cure research requires attending to some particular benefit/risk considerations because the immune systems of infants and very young children are still developing and, thus, may be qualitatively different from adults. Pediatric and adolescent HIV spans the period from neonate to up to 24 years of age in some jurisdictions, encompassing distinct age groups and developmental stages [9]. Arguments for enrolling infants, children, and adolescents in HIV cure trials include the need to prevent delaying the availability of future safe and effective interventions to these groups [123]. However, such an approach is in tension with standard practices of beginning pediatric research only after an intervention has proven to be safe in adults. Further, young people living with HIV might be less likely to be virally suppressed than adults living with HIV due to more frequent disruptions in ART adherence [124]. Limited guidance exists on how to preserve the benefit/risk balance in these populations, and a modified ethics framework may be necessary. Shah argued that, with the exception of research involving very early ART initiation, experimental approaches such as combination regimens may be too risky and speculative to warrant studying in pediatric groups [123, 125]. Consensus appears to have been achieved among experts that ATIs are not recommended for children younger than two years old or those whose HIV infection is resistant to at least two drug classes [9]. Nevertheless, additional deliberation is needed to ensure the benefit/risk balance of HIV cure trials in these younger populations, and acceptable monitoring procedures for them.

### *Informed consent*: attention to language, decision-making, informed consent processes and scientific uncertainty

*Language*: There is a substantial literature on the proper use of language to describe HIV cure research, and those who participate in it [126–132]. Although not specific to HIV cure research, PLWH have advocated for the use of “people-first” language, such as “people living with HIV” rather than “HIV-infected individuals,” and for the use of the word “participants” or “volunteers” rather than “subjects” to describe those actively participating in research [133–135]. While HIV cure research is ultimately directed at “cure”, current research efforts have less ambitious goals and predominately focus on enhancing scientific understanding. Consequently, the word “cure” has also been strongly discouraged from use in informed consent documents and related materials to reduce the possibility of false beliefs that participants will be “cured” from early-phase experiments [9, 17, 136]. Some have recommended the use of the word “experiment” to emphasize the uncertain nature of early-phase research as well as the lack of anticipated direct personal benefit [126].

*Decision-Making*: Few empirical studies have been conducted for the purpose of understanding the decision-making processes of those who accept or decline participation in HIV cure-related trials. However, a longitudinal decision-making study nested within a Thai acute HIV-infection research cohort [74, 137, 138] found that participants’ decisions to undergo an ATI for research purposes were based on their understanding of their body’s likely responses to being off medications [74, 137]. The findings of therapeutic misconception (confusing the intent of the research with clinical care) and therapeutic misestimation (overestimating the probability of direct benefits and underestimating risks) raise ethical concerns in these studies [139, 140]. Of note, therapeutic misconception and misestimation are commonly experienced by participants of early phase trials, and are more ethically problematic than therapeutic optimism (the hope for positive outcomes) [139, 141]. Similarly, surveys conducted in the ACTG 5366 trial testing a latency-reversing agent and the ACTG 5345 study interrogating biomarkers of viral rebound found that approximately 20% of participants inaccurately reported believing that the trial did not contain any clinical risk [116, 118]. Despite such efforts to measure participants’ understanding during biomedical HIV cure trials [74], there is a lack of consensus on the best way to assess the extent of therapeutic misconception and misestimation, let alone reduce them. Moreover, concerns regarding therapeutic misconception and misestimation may be heightened in low- and middle-income countries (LMICs), particularly in places where research literacy levels are lower and where unproven, questionable HIV “cures” have flooded informal markets for decades [142–144].

Additionally, how HIV clinicians perceive and communicate aspects of HIV cure trials to patients is unclear [88, 145, 146]. Due to the chronic nature of HIV infection, many PLWH have long-established, trusting relationships with particular clinicians, and these relationships often function as decision-making units when deciding whether to join a clinical trial [147].

*Informed Consent Processes*: Concerns have been raised around the clarity, specificity, and consistency of text used in informed consent forms [17]. A 2014 review of 13 HIV cure-related informed consent forms by Henderson revealed inconsistencies around the descriptions of study aims, risks, and benefits (or lack thereof) of the research, with conflicting messages about the nature versus the likelihood of direct clinical benefits to study participants. Based on their analysis, the authors recommended surrogate endpoints (e.g., reduction in HIV reservoir size) not be portrayed as possible clinical benefits [17].

In a separate review, Bromwich and colleagues outlined key informed consent challenges for HIV cure research: (1) how trial information is communicated to potential participants, (2) whether potential participants fully understand key features of the clinical trials in which they are being asked to participate, and (3) the degree to which potential study participants’ motivations to enroll in low-benefit/high-risk research are altruistically motivated [18]. Overall, it seems clear that empirical research and further deliberation is needed to strengthen and improve the quality of informed consent in general [148–150].

*Scientific Uncertainty*: The ethical implications of scientific uncertainty have received limited attention in the HIV cure research field [24, 151, 152]. Although risk and uncertainty imply a lack of knowledge about future outcomes, risk refers to the probability and magnitude about possible harms, whereas uncertainty refers to the lack of predictability due to insufficient scientific evidence [153]. Early-phase research inherently carries more scientific uncertainty than later-phase research [154].

HIV cure research is fraught with uncertainty, as evidenced by the cases of the Boston patients (who received hematopoietic stem cell transplants) [155, 156] and the Mississippi child (who received early ART administration soon after birth) [157, 158]. Despite initial beliefs of cure, the Boston patients and Mississippi child experienced viral rebound following periods of undetectability ranging from several months (Boston patients) to more than two years (Mississippi child) after ART was interrupted.

Scientific uncertainty is particularly relevant during the informed consent process around descriptions of ATIs. ATIs involve periods of unpredictable and stochastic rebounds of virus that no currently known biomarker can accurately predict [9]. Further, the unknown risks of experimental interventions, as well as how results compare between animal models and human testing, present additional uncertainties [45, 93, 159]. Factors, such as small sample sizes, observational study designs versus randomized controlled trials, and varying immunological, virologic, and clinical monitoring strategies, compound scientific uncertainties in the field [115]. Thus, it may be useful to assess ways to explicitly discuss and consider scientific uncertainty during the informed consent process (e.g., including a separate section on scientific uncertainty in informed consent documents). Additional information about how uncertainty is communicated to, and understood by, participants and the public is also needed.

### *Respect for enrolled participants and community*: perspectives of PLWH and affected communities and representation

*Perspectives of PLWH and Affected Communities*: Much HIV cure research centers on the biomedical aspects of cure, while far less attention has been placed on the psychosocial context of those for whom a cure is being sought [160]. There has been limited research evaluating the motivations, perceptions, needs, concerns, desires, tensions, and experiences of PLWH who participate in these trials [70, 160]. A comprehensive understanding of how PLWH view and experience these innovative strategies is ethically essential because it preserves respect for affected communities [45]. Sparked by the 2014 FDA Patient-Focused Drug Development Initiative [161], and the ensuing *Voice of the Patient* report for HIV cure research [162], greater weight and appreciation has been given to PLWH’s preferences in developing novel HIV therapeutic options [163–165]. Implementing patient-centered research is particularly important given increased focus on health-related quality of life and psychosocial well-being for PLWH [166].

Incorporating the participant perspective in research can be facilitated through robust interdisciplinary research by integrating bioethicists and socio-behavioral researchers in trial teams [70, 118, 167]. Empirical ethics and socio-behavioral research can help inform prioritization decisions and determining the acceptability of HIV cure research interventions, which may differ in association with various demographic characteristics [23, 45]. For example, focus groups conducted in the United States revealed acceptability concerns for somatic HIV cure cell and gene therapy among PLWH, particularly among ethnic and racial minorities [168]. A discrete choice experiment conducted in Europe among 150 PLWH and 160 HIV care providers revealed that acceptability would increase if clinical risks of cell and gene therapy could be minimized [122]. As HIV cure clinical trials get implemented globally, we will need to better understand perspectives of PLWH and affected communities in different contexts.

*Community Representation*: Ultimately, all stakeholders, including patients, providers, government and overall community acceptance will be critical to the success of any HIV cure regimen [28, 32, 34, 35, 45, 70, 169]. Although good participatory practice guidelines exist for biomedical HIV prevention research [170], and have been adopted for tuberculosis [171] and emerging pathogens [172], these guidelines may not be seamlessly extrapolated to early-phase HIV cure trials. Additional guidance is needed to inform the ethics of engaging communities around ATIs, HIV transmission risk, and relevant standards of care and prevention. Importantly, as discussed above, the science, language, and messaging around HIV cure research remain extremely complex and technical. Participants and communities must be engaged as mutually respected and integral partners in the research enterprise [6, 133, 173]. Communication should be culturally sensitive, prompt, and easily understandable. Community input should be sought and utilized in defining research priorities and decision-making processes. Efforts should also be taken to build meaningful, long-term relationships with relevant communities regarding the goals of HIV cure research, and not simply around specific short-term trials.

## Conclusions

HIV cure research ethics has an unfinished agenda, which will require further inquiry and deliberation. Scientific research and bioethics should work in tandem to advance ethical HIV cure research [174]. While relying on established ethical guidelines, the field must work towards careful use of language, managing expectations, and high-quality informed consent. HIV cure research must have an acceptable benefit/risk balance and account for scientific uncertainty, particularly before interrupting ART and during ATIs. As the science evolves, it will be essential to better understand the perspectives of PLWH and of affected communities to ensure respect of participants, the continuation of successful research efforts and social value. Ethical considerations will need to be grounded in the reality of ongoing trials and local contexts. Researchers conducting clinical trials should make a genuine commitment to meaningful community and stakeholder engagement. Because the science of HIV cure research will continue to rapidly advance, ethical considerations should be revisited and refined. Sufficient financial and human resources should be dedicated to resolving these critical challenges.

## Data Availability

Not applicable.

## Abbreviations

ACTG: AIDS Clinical Trials Group
AE: Adverse event
ART: Antiretroviral treatment
ATI: Analytical treatment interruption
AVRC: AntiViral Research Center
CAB: Community advisory board
CAPS: Center for AIDS Prevention Studies
CARE: Collaboratory of AIDS Researchers for Eradication
CSS: Community Scientific Subcommittee
DARE: Delaney AIDS Research Enterprise
FDA: Food and Drug Administration
H+ARP-PS: HIV + Aging Research Project-Palm Springs
HIV: Human immunodeficiency virus
IRB: Institutional review board
LMIC: Low- and middle-income country
NIH: National Institutes of Health
PLWH: People living with HIV
PrEP: Pre-exposure prophylaxis
SARS-CoV-2: Severe Acute Respiratory Syndrome-Coronavirus 2
