# Correction: Ethics of HIV cure research: an unfinished agenda

**DOI:** 10.1186/s12910-025-01362-7

**Published:** 2026-02-19

**Authors:** Karine Dubé, John Kanazawa, Jeff Taylor, Lynda Dee, Nora Jones, Christopher Roebuck, Laurie Sylla, Michael Louella, Jan Kosmyna, David Kelly, Orbit Clanton, David Palm, Danielle M. Campbell, Morénike Giwa Onaiwu, Hursch Patel, Samuel Ndukwe, Laney Henley, Mallory O. Johnson, Parya Saberi, Brandon Brown, John A. Sauceda, Jeremy Sugarman

**Affiliations:** 1 University of North Carolina at Chapel Hill, Gillings School of Global Public Health, 4108 McGavran Greenberg Hall, Chapel Hill, NC 27599-7469 USA; 2 HIV + Aging Research Project – Palm Springs (HARP–PS), Palm Springs, CA USA; 3 AntiViral Research Center (AVRC) Community Advisory Board (CAB), San Diego, CA USA; 4 Collaboratory of AIDS Researchers for Eradication (CARE) CAB, Chapel Hill, NC USA; 5 AIDS Action Baltimore, Baltimore, MD USA; 6 Delaney AIDS Research Enterprise (DARE) Community Advisory Board (CAB), San Francisco, CA USA; 7 BEAT-HIV Collaboratory CAB, Philadelphia, PA USA; 8 defeatHIV CAB, Seattle, WA USA; 9 AIDS Clinical Trials Group (ACTG) Community Scientific Subcommittee (CSS) Ethics Working Group, Nationwide, Birmingham, USA; 10 AIDS Clinical Trials Group Global CAB, Washington, DC USA; 11 Institute of Global Health and Infectious Diseases HIV Treatment and Prevention CAB, University of North Carolina at Chapel Hill, Chapel Hill, NC USA; 12 Charles R. Drew College of Medicine and Science, Los Angeles, CA USA; 13 Center for the Study of Women, Gender, and Sexuality (School of Humanities), Rice University, Houston, TX USA; 14 UCSF, San Francisco, CA USA; 15 Department of Social Medicine, Population and Public Health, Center for Healthy Communities, University of California, Riverside, CA USA; 16 Johns Hopkins Berman Institute for Bioethics, Baltimore, MD USA

**Correction to: BMC Medical Ethics (2021) 22:83**

**https://doi.org/10.1186/s12910-021-00651-1**

In this article [1], the given and family name of the co-author Morénike Giwa Onaiwu was incorrectly structured. The name of the author was displayed correctly in all versions at the time of publication.

The original article has been corrected.
